# Melorheostosis with renal arterio-venous malformation: A case report with review of literature

**DOI:** 10.4103/0971-5851.56336

**Published:** 2009

**Authors:** Abdul Rashid Lone, Mushtaq Ahmad, Sheikh Aejaz Aziz, Gul Mohammad Bhat, Javid Rasool Bhat, Rifat Jahan, Shoukat H Khan

**Affiliations:** *Department of Medical Oncology, RCC, Sheri Kashmir Institute of Medical Sciences, Srinagar, Kashmir, India*; 1 *Department of Clinical Hematology, RCC, Sheri Kashmir Institute of Medical Sciences, Srinagar, Kashmir, India*; 2 *Department of Nuclear Medicine, RCC, Sheri Kashmir Institute of Medical Sciences, Srinagar, Kashmir, India*

**Keywords:** *Flowing periosteal hyperostosis*, *Leri′s disease*, *melorheostosis*, *renal AV malformation*

## Abstract

Melorheostosis, also known as Leri′s disease and flowing periosteal hyperostosis, is a rare cause of pain and stiffness in a limb. The appearance is of "candle greasing" down one side of one or several bones of the body. We describe a case referred to tertiary care center with suspicion of renal cell carcinoma with diffuse bone metastasis. After reassessment, the patient was diagnosed melorheostosis with renal AV malformation. He was reassured about the benign nature of the disease and is asymptomatic.

## INTRODUCTION

Melorheostosis is a rare nongenetic developmental anomaly, first described in 1922 by Leri and Joanny. It can present at any age, and the diagnosis is made by radiography. This skeletal disorder is due to a disturbance of both intramembranous and endochondral bone formations and therefore belongs to a group of "mixed sclerosing bone dysplasias." The prognosis is uncertain; and treatment, symptomatic. Numerous soft-tissue and vascular anomalies have been reported in patients with melorheostosis.

## CASE REPORT

**Figure 1:**
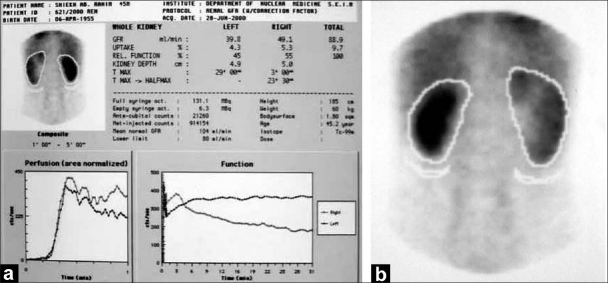
(a) Tc-99m DTPA renogram (PA). Tc-99m DTPA renogram (AP); (b) Tc-99m DTPA renogram (AP)

**Figure 2:**
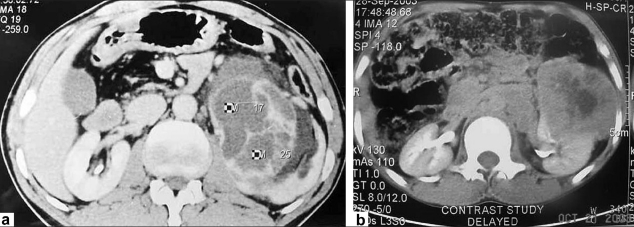
CECT (a,b) revealed a large multiloculated cystic mass arising from left kidney, impinging upon the pelvis and causing hydronephrosis

**Figure 3:**
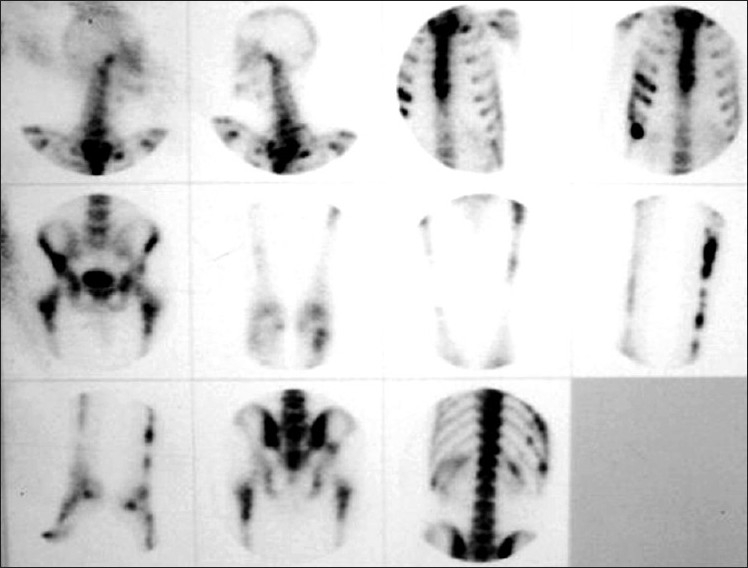
TC-99m MDP bone scan

**Figure 4:**
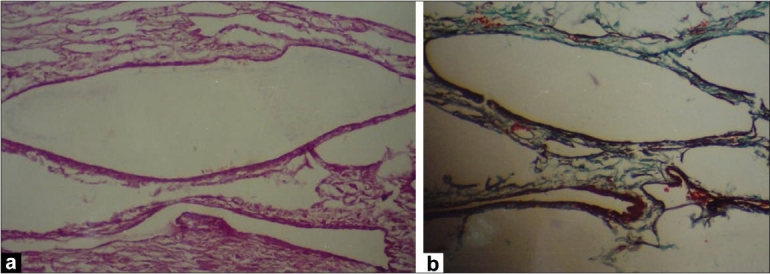
(a) Arteriovenous anomaly: Tangle of abnormal muscularized arteries and ecstatic veins without intervening capillaries (H and E, ×400); (b) Masson's trichrome showing abnormal muscularized and fibrotic arterial walls (MTC, ×400)

**Figure 5:**
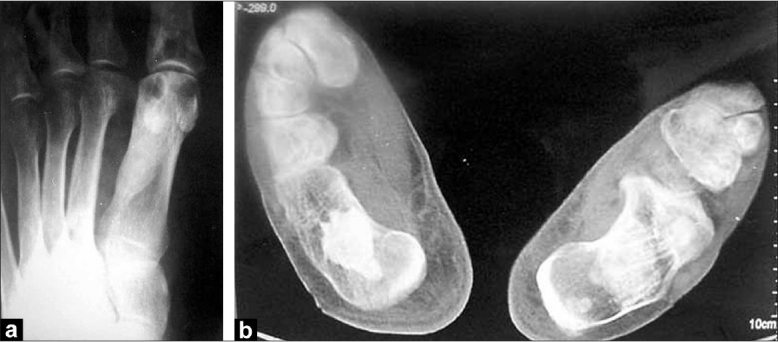
(a, b) Irregular, hyperosteotic changes of cortex, resembling melting wax dripping on the side of a candle. Dense sclerotic linear areas are seen, mainly in the cortex

A 42-year-old male patient presented in 2001 with urinary symptoms. His physical examination was unremarkable, with normal pulse and blood pressure. Investigation at presentation revealed a normal hemogram and erythrocyte sedimentation rate (ESR). Fasting blood sugar was 90 mg/dL (normal, 90-110 mg/dL), and kidney functions were normal. Ultrasonography revealed grade one benign prostatic hyperplasia with multiple cysts in left kidney. Isotopic Renogram done after intravenous injection of 5 milli Curies of Technetium-99m diethylene triamine penta acetic acid (Tc-99m DTPA) under a large field of view Gamma camera revealed a relative renal function of 45% in left kidney and 55% in the right kidney [Figure 1]. The overall scan impression was that of an enlarged left kidney with mild functional impairment. On reevaluation in 2003, his physical examination was unremarkable. Investigations revealed a normal hemogram, normal results of kidney function tests and normal levels of serum and urinary calcium. Parathormone (PTH) levels were within normal limits. Intravenous pyelogram (IVP) revealed a well-defined cystic lesion in the left kidney near pelvicalyceal system causing splashing of pelvis, obstructed calyces and caliectasis. Contrast-enhanced CAT scan of abdomen (CECT) revealed large multiloculated cystic mass arising from left kidney, impinging on pelvis and causing hydronephrosis [Figure 2]. Fine-needle aspiration was inconclusive. Tc-99m Methylene Diphosphonate (MDP) bone scan revealed abnormal tracer uptake in multiple ribs, femur, pelvis, foot bones, tibia and 9^th^, 10^th^ and 11^th^ dorsal vertebrae [Figure 3]. Hemogram and serum chemistry were normal. Left radical nephrectomy was done in April 2003. Operative findings revealed a mass involving left kidney, with hydronephrotic changes. Histopathological examination (HPE) revealed arterial malformation of kidney [Figure 4]. Patient was referred to tertiary care center with an impression of renal cell carcinoma with diffuse bone metastasis. He was reevaluated and found to have high serum alkaline phosphatase (Alp) levels. In view of the long history, good performance status and histopathological examination, skeletal survey was done, which revealed hyperostosis of long bones, and the bone of foot resembling wax dripping on one side [Figure 5]. A final diagnosis of melorheostosis was made and the patient reassured. He is on our follow-up for the last 5 years and is asymptomatic.

## DISCUSSION

Melorheostosis is a rare nongenetic developmental anomaly. The name is derived from the Greek words *melos,* meaning limb; and *rhein,* meaning to flow. Its etiology is unknown, patients present at any age and both sexes are affected equally. It affects mainly the long bones; but in some cases, also short bones of the hands and feet; and rarely, the axial skeleton. [1,2] The appearance is of "candle greasing" down one side of one or several of the bones of one half of the body.[3] Although changes mainly affect cortex, sclerotic changes may extend into the spongiosa of bones. Bone scintigraphy is positive and shows moderately increased tracer uptake.[4 The skeletal disorder is due to a disturbance of both intramembranous and endochondral bone formations and therefore belongs to the group of "mixed sclerosing bone dysplasias," a classification proposed by Greenspan.[5,6] Melorheostosis is found to be associated with anomalies of blood or lymph vessels in 5% to 17% of cases. [7,8] Remarkably vascular anomalies have been always reported to be ipsilateral and have included capillary dysplasia, vascular nevi, arteriovenous shunts, varices, lymphactesia, aneurysms and glomangiosomas, renal artery stenosis, aortic valve insufficiency. [8-12] 

The etiology and pathogenesis of melorheostosis are unknown. Two major hypotheses exist: First, the bony lesions have been ascribed to sclerotomes, which are areas of sensory innervations of skeleton[13] and second, the bony lesions are proposed to originate from a postzygotic mutation occurring during embryogenesis.[14,15] Histopathology of the bone specimen reveals nonspecific hyperostotic, mainly lamellar, bone formation.[16] 

Isolated cases of malignancy have been reported in association with melorheostosis - one case of osteosarcoma and one of malignant fibrous histiocytoma. At least, one death has been reported due to complications of this disease, due to resistant pleural effusion secondary to associated vascular malformation. The clinical course is slowly progressive. Severe symptoms may require treatment by sympathectomy or even amputation.[17]
